# Undetected pituitary adenoma in a patient with retinitis pigmentosa

**DOI:** 10.1186/s41016-019-0168-5

**Published:** 2019-08-13

**Authors:** Mousa Taghipour, Nima Derakhshan, Arash Saffarian, Meisam Ghanbari

**Affiliations:** 1 Neurosurgery Department, Shiraz University of Medical Sciences, Shiraz, Iran; 2 Poostchi Ophthalmology Research Center, Shiraz University of Medical Sciences, Shiraz, Iran

**Keywords:** Sellar mass, Retinitis pigmentosa, Pituitary adenoma, Vision loss, Tunnel vision

## Abstract

**Background:**

Retinitis pigmentosa (RP) is one of the most severe hereditary retinal disorders with a worldwide prevalence reaching 1 in every 3000–5000 people and a total of almost one million affected individuals. RP is heterogeneous in its clinical presentations but typically presents as progressive visual dysfunction, including nyctalopia in adolescence, restricted peripheral vision (tunnel vision) in young adults, and loss of central vision at an advanced age.

**Case description:**

Herein, we want to report a case of RP who presented with gradual worsening of vision and headache, and further evaluation revealed a concomitant non-functional pituitary macroadenoma. Ophthalmologic evaluation revealed a little chance for him to regain his vision, so the patient refused to undergo endoscopic surgical resection. However, he is still under clinic-radiologic follow-up, to be evaluated for progression in tumor size and obstructive hydrocephalus.

**Conclusion:**

Presenting with similar symptoms of tunnel vision, the simultaneous occurrence of these two diseases in a patient may delay the diagnosis of the latter, leading to its progression.

## Background

Retinitis pigmentosa (RP) is one of the most severe hereditary retinal disorders with a worldwide prevalence reaching 1 in every 3000–5000 people [1] and a total of almost one million affected individuals. RP is heterogeneous in its clinical presentations but typically presents as progressive visual dysfunction, including nyctalopia in adolescence, restricted peripheral vision (tunnel vision) in young adults, and loss of central vision at an advanced age. More than 45 genes have been identified to cause the retinal disease through different inheritance patterns autosomal-recessive (50–60%), autosomal-dominant (30–40%), or X-linked (5–15%) [2].

RP usually begins with the involvement of rod photoreceptors followed by secondary degeneration of cone photoreceptor. Typical funduscopic findings include bone-spicule retinal pigmentation (initially in the peripheral retina), attenuation of retinal vessels, waxy pallor of the optic disc, and various degrees of chorioretinal atrophy.

Treatment is directed at slowing down the degenerative process by vitamin therapy and sunlight protection plus treating ophthalmologic complications (cataract and macular edema) and vision rehab. Future therapeutic prospects include retinal prosthesis [3] as well as gene therapy [4].

On the other hand, pituitary adenomas are a common type of brain tumors comprising 15% of all intracranial primary brain tumors. Pituitary adenomas may be clinically silent or may present as three categories of manifestations. The first category in clinical presentation is seen with functional (hormonally active) adenomas which present as a certain endocrinopathy, due to excess of a certain hormone. The examples include acromegaly, Cushing disease, prolactinoma, secondary hyperthyroidism due to excess of growth hormone, adrenocorticotropin-stimulating hormone, prolactin, and thyroid-stimulating hormone, respectively. The second category of clinical presentations are seen with nonfunctional adenomas; these adenomas are not accompanied with secreting excess amounts of a certain hormone though have the potential to be diagnosed at a later stage and grow to larger sizes and produce symptoms of mass effect, i.e., headache, visual field restrictions (typically produce tunnel vision due to pressure on optic chiasma), cranial neuropathies, and symptoms of raised intracranial pressure. The third category of clinical presentation of a pituitary adenoma is apoplexy, which presents itself with symptoms of severe sudden-onset headache and sudden visual loss followed by decreased production of pituitary hormones.

Presenting with similar symptoms of tunnel vision, the simultaneous occurrence of these two diseases in a patient may delay the diagnosis of the latter, leading to its progression.

Herein, we want to report a case of RP who presented with gradual worsening of vision and headache, and further evaluation revealed a concomitant non-functional pituitary macroadenoma.

## Case presentation

**Fig. 1:**
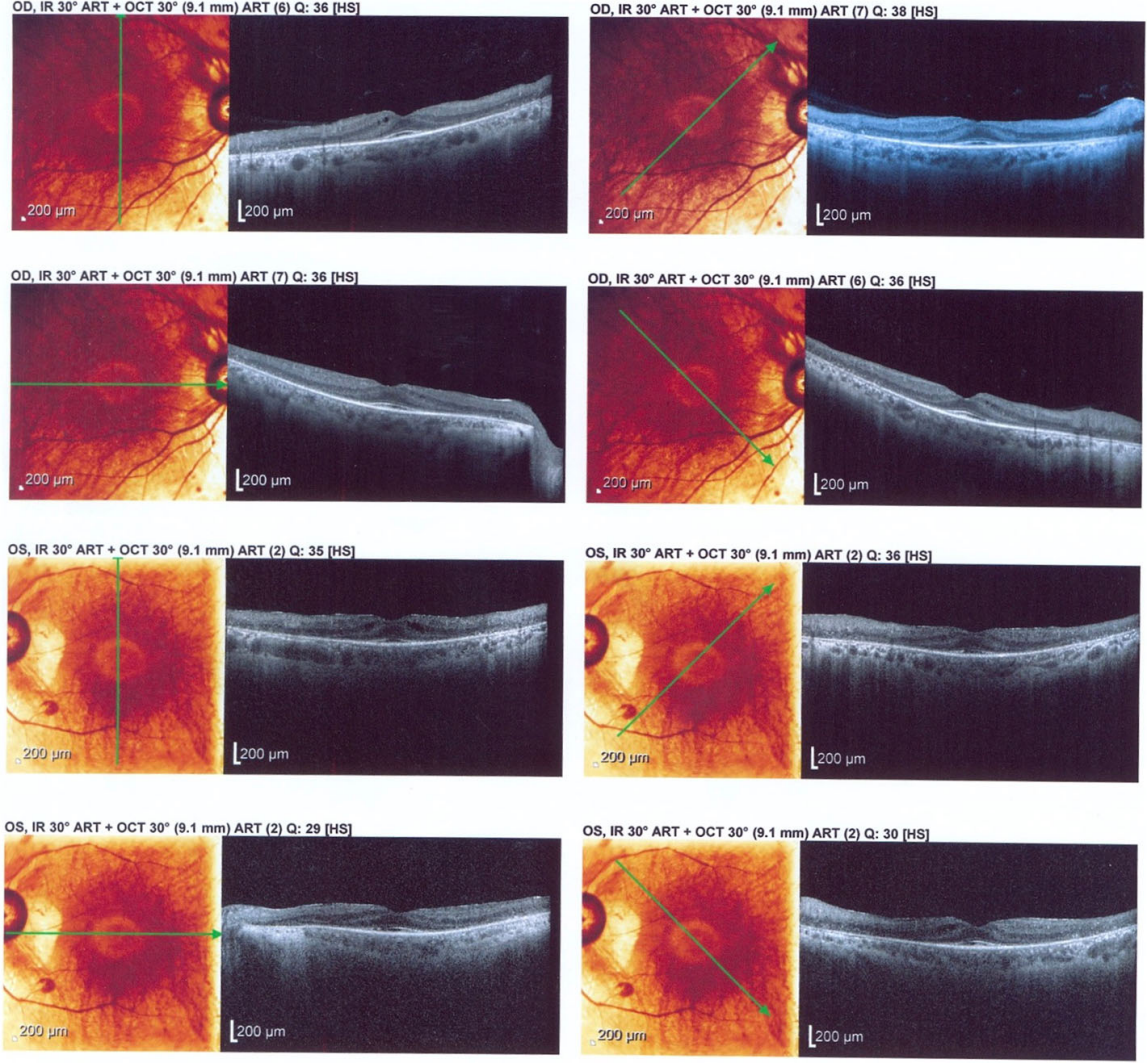
OCT of the macula shows a diffuse loss of ellipsoid zone, sparing the fovea in both eyes. This finding is suggestive of diffuse photoreceptor damage with preservation of foveal cones. There is no cystoid macular edema, neither intraretinal or subretinal fluid accumulation. Choroidal thickness is normal

**Fig. 2:**
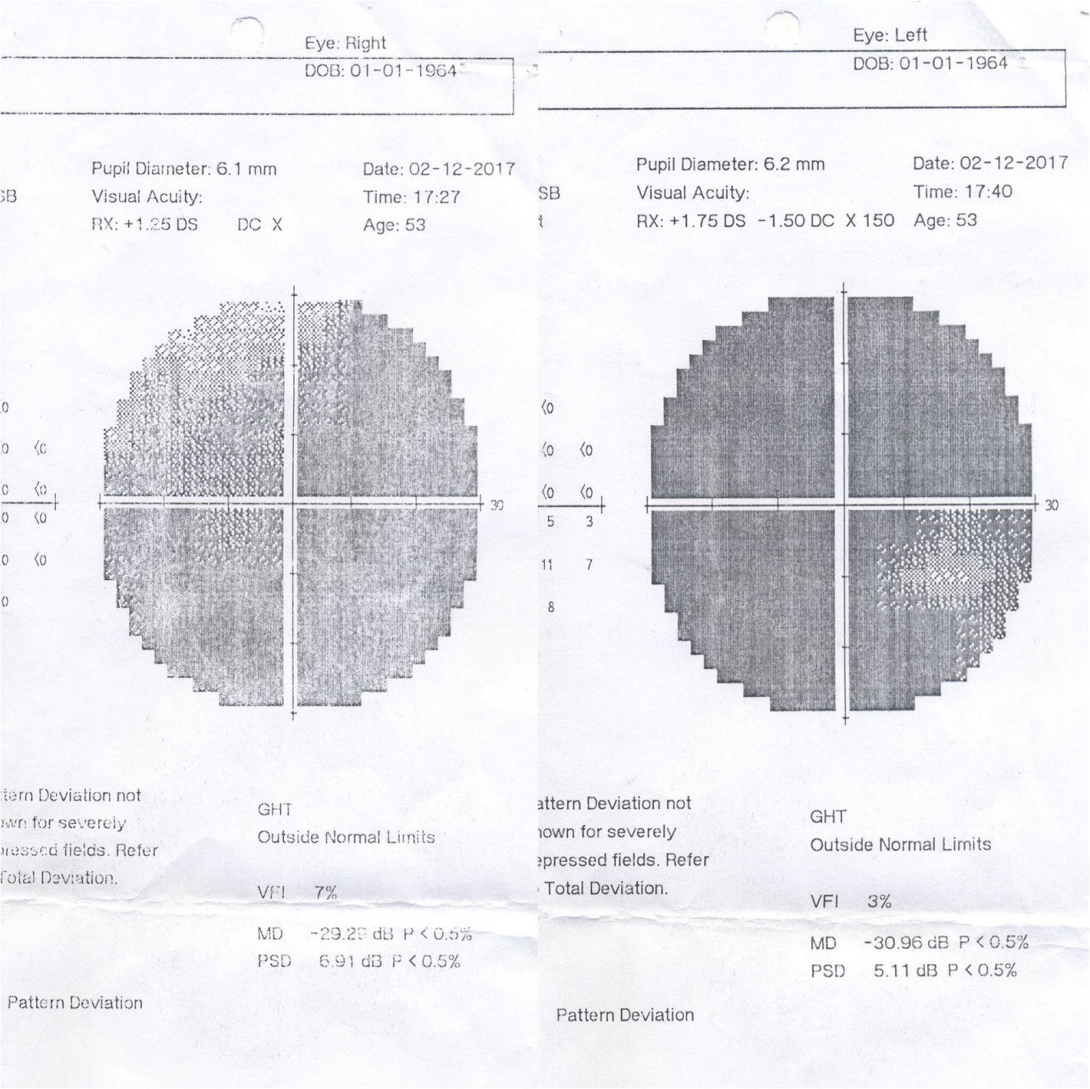
Perimetry showing a very severe loss of visual field in both eyes

A 59-year-old man, a former captain of the army, who was a known case of RP in the last 40 years was referred by an ophthalmologist to our neuro-oncology clinic with symptoms of headache, progression of visual dysfunction including further restriction in visual field, and severe decrease in visual acuity within the last 2 years. On physical examination, he could barely do the finger count from a 1-m distance in both eyes and the visual field was severely restricted to a small portion of central field. Funduscopic examination revealed the typical findings of RP. He had a relative afferent pupillary defect in the right eye (Marcus Gunn pupil) and both pupils had a sluggish response to light. The rest of his neurologic examination was unremarkable. Optical coherence tomography (OCT) of the macula showed a diffuse loss of ellipsoid zone, sparing the fovea (Fig. 1). Perimetry demonstrated a severe visual field restriction in both eyes which interestingly affected the temporal fields of both eyes rather than the nasal fields (Fig. 2).

**Fig. 3:**
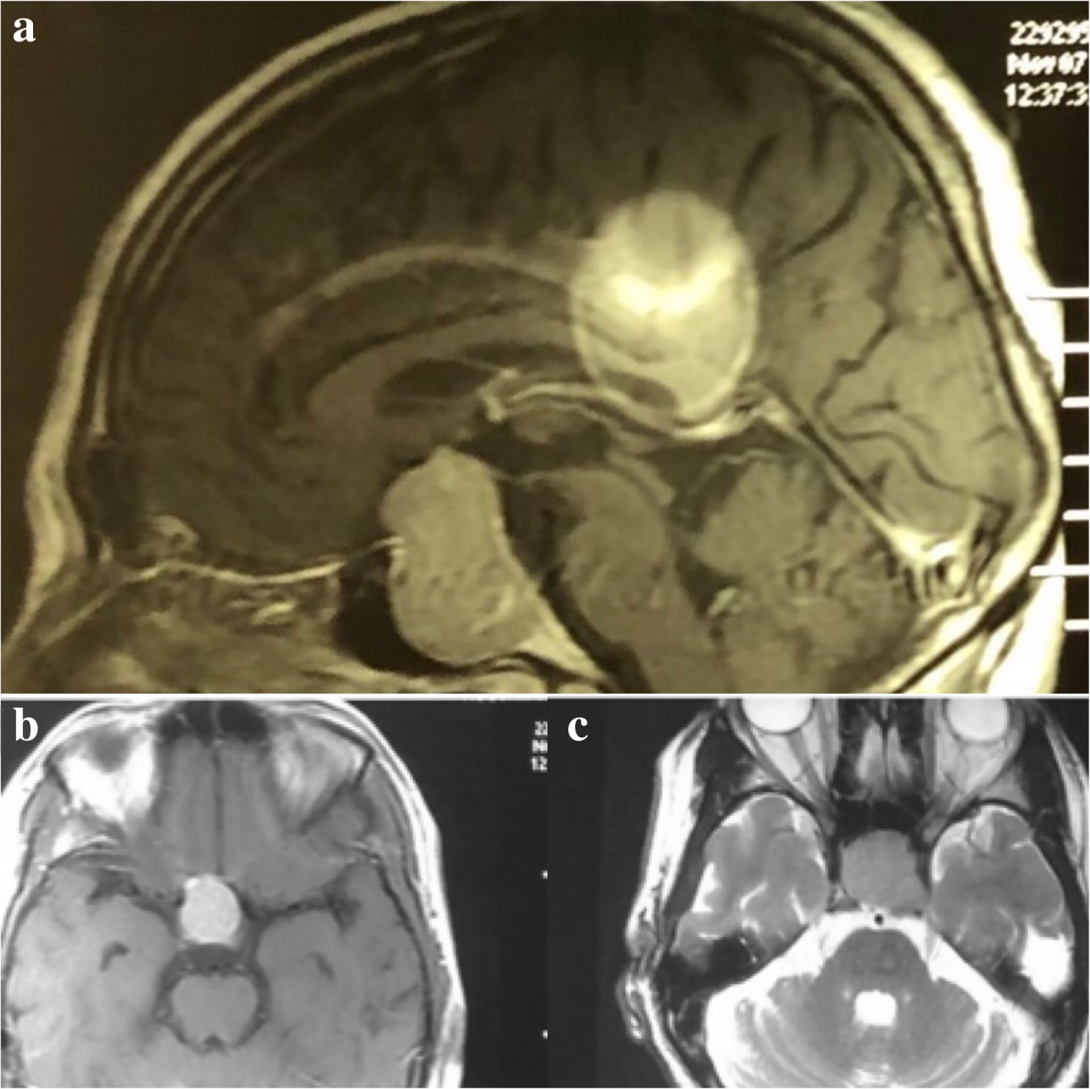
Brain MRI. **a** Post-contrast sagittal T1-weighted and **b** axial T1-weighted images showing homogenous enhancement of sellar lesion in favor of a pituitary macroadenoma. In the **a** image, a metallic artifact is visible. **c** Isointense sellar lesion in T2-weighted acquisition

Due to the presence of headache, a right-sided Marcus Gunn pupil and further restriction of temporal fields in perimetry, a brain CT scan was requested which revealed a sellar lesion, resembling a pituitary adenoma. So, he was advised to perform a gadolinium-enhanced magnetic resonance imaging (MRI) and also hormonal tests. The lab results for hormonal assay were all in normal range, depicting a non-functional adenoma. Due to the presence of an intracranial shell fragment, radiology technicians canceled the MRI and he refused to return to neuro-oncology clinic for 2 months. After this time, he was visited again and considering the safety of brain MRI in certain intracranial ballistics (non-steel-containing) [5] and also the benefits of the correct diagnosis outweighing the potential risks in this very patient, we advised the patient to undergo the brain MRI after obtaining the informed consent. Brain MRI, despite showing a metallic artifact, revealed a sellar lesion with hypo-intensity in T1, iso- to hyper-intensity in T2 and homogenous enhancement after gadolinium injection, all in favor of a pituitary macroadenoma (Fig. 3).

Another reason that led to his sellar mass remaining undetected was the presence of an intracranial ballistic fragment. He was serving as an Iranian Army soldier during the Iran-Iraq war and suffered a penetrating intracranial retained ballistic fragment when he was 22.

We offered endoscopic trans-nasal trans-sphenoidal surgery for the removal of the sellar lesion, but the patient who was being informed that his vision would not change very much by three retina fellowships decided against this operation and was discharged.

We also offered him the options of stereotactic biopsy followed by radiotherapy and stereotactic radio-surgery. However, considering the dismal prognosis of acquiring visual improvement followed by either treatment, he refused other therapeutic options and follow-up MRI studies as well. The patient is still being followed on a bi-monthly basis (8-month follow-up). His brain CT scan has not shown any obvious change in size of lesion and no obstructive hydrocephalus during this period, and he is still under follow-up to be evaluated for these two parameters, as well as clinical symptoms of high intracranial pressure.

## Discussion and conclusion

The association between a non-functional pituitary adenoma and RP was not previously described in the literature. However, a few reports are present regarding pigmentation of retina in acromegalics, resembling RP [6, 7]. Pituitary eosinophilic adenoma and acromegaly may also co-exist with RP in Laurence-Moon-Biedl syndrome [8].

The similar clinical presentation of both conditions with “tunnel vision” (peripheral visual field defects) and attributing the worsened vision to the progression of RP was the main reason for late diagnosis of pituitary adenoma in this very patient.

Although the natural history of visual dysfunction is known to be progressive in individuals with RP, patients who experience accelerated visual field loss, headaches, and lateralizing neurologic deficits should undergo neuroimaging to rule out a concomitant neurosurgical problem.

The decision to operate such patients who suffer concomitant retinal disease and pituitary adenoma should encounter measurement of retinal nerve fiber layer (RNFL) thickness via OCT. Recent evidence shows that RNFL thickness of the temporal quadrant is a reliable predictor of visual recovery following trans-sphenoidal surgery [9].

Patients with concomitant pituitary adenoma and ophthalmologic comorbidity pose as a clinical dilemma for neurosurgeons and neuro-ophthalmologists. Pre-operative assessment of the retina with OCT and the optic tract via visual pathway diffusion tensor imaging (DTI) tractography are helpful adjuncts for such a multi-disciplinary decision-making [10].

## Data Availability

The datasets used and patient’s information during the current study are available from the corresponding author on request by the Editor-in-Chief of this journal.

## Abbreviations

DTI: Diffusion tensor imaging
MRI: Magnetic resonance imaging
OCT: Optical coherence tomography
RFNL: Retinal nerve fiber layer
RP: Retinitis pigmentosa

## References

[1] Kurata K, Hosono K, Hotta Y (2018). Clinical and genetic findings of a Japanese patient with RP1-related autosomal recessive retinitis pigmentosa. Doc Ophthalmol.

[2] Hartong DT, Berson EL, Dryja TP (2006). Retinitis pigmentosa. Lancet.

[3] Chow AY, Chow VY, Packo KH, Pollack JS, Peyman GA, Schuchard R (2004). The artificial silicon retina microchip for the treatment of visionloss from retinitis pigmentosa. Arch Ophthalmol.

[4] Hamel C (2006). Retinitis pigmentosa. Orphanet J Rare Dis.

[5] Eshed I, Kushnir T, Shabshin N, Konen E (2010). Is magnetic resonance imaging safe for patients with retained metal fragments from combat and terrorist attacks?. Acta Radiol.

[6] Erem C, Ersöz HÖ, Ukinç K, Avunduk AM, Hacihasanoglu A, Koçak M (2006). Acromegaly presenting with diabetic ketoacidosis, associated with retinitis pigmentosa and octreotide-induced bradycardia. Endocrine.

[7] Cosemans I, Demaerel P, Wets B, De Hauwere B, Spileers W (1999). Retinitis pigmentosa in association with acromegaly: a case report. Doc Ophthalmol.

[8] Hiraki S, Tsutsumi A, Tsuroi S (1971). An autopsy case of Laurence-Moon-Biedl syndrome with pituitary eosinophilic adenoma. Pathol Int.

[9] Kawaguchi T, Ogawa Y, Tominaga T (2019). Retinal nerve fiber layer thickness measurement for predicting visual outcome after transsphenoidal surgery–optic disc atrophy is not the deciding indicator. World Neurosurg.

[10] Hajiabadi M, Alimohamadi M, Fahlbusch R (2015). Decision making for patients with concomitant pituitary macroadenoma and ophthalmologic comorbidity: a clinical controversy. World Neurosurg.

